# Integrative Genomics: Quantifying Significance of Phenotype-Genotype Relationships from Multiple Sources of High-Throughput Data

**DOI:** 10.3389/fgene.2012.00202

**Published:** 2013-05-31

**Authors:** Eric R. Gamazon, R. Stephanie Huang, M. Eileen Dolan, Nancy J. Cox, Hae Kyung Im

**Affiliations:** ^1^Department of Medicine, University of ChicagoChicago, IL, USA; ^2^Department of Human Genetics, University of ChicagoChicago, IL, USA; ^3^Department of Health Studies, University of ChicagoChicago, IL, USA

**Keywords:** eQTLs, FDR, gene expression, genomics, GWAS, integrative genomics, permutation, phenotype

## Abstract

Given recent advances in the generation of high-throughput data such as whole-genome genetic variation and transcriptome expression, it is critical to come up with novel methods to integrate these heterogeneous datasets and to assess the significance of identified phenotype-genotype relationships. Recent studies show that genome-wide association findings are likely to fall in loci with gene regulatory effects such as expression quantitative trait loci (eQTLs), demonstrating the utility of such integrative approaches. When genotype and gene expression data are available on the same individuals, we and others developed methods wherein top phenotype-associated genetic variants are prioritized if they are associated, as eQTLs, with gene expression traits that are themselves associated with the phenotype. Yet there has been no method to determine an overall *p*-value for the findings that arise specifically from the integrative nature of the approach. We propose a computationally feasible permutation method that accounts for the assimilative nature of the method and the correlation structure among gene expression traits and among genotypes. We apply the method to data from a study of cellular sensitivity to etoposide, one of the most widely used chemotherapeutic drugs. To our knowledge, this study is the first statistically sound quantification of the overall significance of the genotype-phenotype relationships resulting from applying an integrative approach. This method can be easily extended to cases in which gene expression data are replaced by other molecular phenotypes of interest, e.g., microRNA or proteomic data. This study has important implications for studies seeking to expand on genetic association studies by the use of omics data. Finally, we provide an R code to compute the empirical false discovery rate when *p*-values for the observed and simulated phenotypes are available.

## Introduction

The availability of genome-wide datasets is facilitating unprecedented insights into various aspects of cellular processes. Technological advances (Metzker, 2010) in high-throughput methods are contributing to new approaches in genomics, transcriptomics (Wang et al., 2009), proteomics (Farnham, 2009), and epigenomics (Laird, 2010; Zhou et al., 2011), allowing in-depth interrogation of diverse biological processes. A primary challenge from the tremendously heterogeneous and increasingly massive datasets is data integration – a challenge that is inevitably bound to intensify with the deluge of these high-throughput datasets. Nevertheless, among the many exciting promises, integrative approaches are likely to yield a comprehensive map of genome function (Degner et al., 2012) as well as a high-resolution view into the complex logic of biological systems (Hawkins et al., 2010).

Indeed, while genome-wide association studies (GWAS) have identified thousands of common genetic variants associated with diseases and other complex human traits (Hindorff et al., 2009), functional understanding of many of the variants remains elusive. Integrating other omics datasets into genome-wide analyses offers the potential to provide systematic insight into the mechanisms underlying the observed genotype-phenotype relationships. One common approach to the integration of functional data into GWAS is the use of expression quantitative trait loci (eQTL; Stranger et al., 2007a; Duan et al., 2008; Schadt et al., 2008) information to expand on the nature of the genetic component to complex phenotypes (Gamazon et al., 2010a; Nicolae et al., 2010). Such an integrative approach is clearly extensible to the use of protein (Garge et al., 2010) or microRNA quantitative trait loci (Gamazon et al., 2012), indeed other functionally relevant features of the genome, to improve identification of functional variants.

Our group (Huang et al., 2007a; Welsh et al., 2009; Nicolae et al., 2010) and others (Cheung et al., 2003; Correa and Cheung, 2004; Stranger et al., 2007b; Nica et al., 2010) have used the HapMap lymphoblastoid cell lines (LCLs) as a model for human genotype-phenotype relationships. The cell lines have been the subject of several whole-genome gene expression profiling studies (Montgomery et al., 2010; Pickrell et al., 2010; Stranger et al., 2012) to identify functional loci (e.g., eQTLs) with potentially important links to SNP associations emerging from genome-wide studies. Furthermore, the cell lines have been utilized to identify the molecular consequences associated with various exposures (Dermitzakis, 2012), such as drugs (Huang et al., 2007b), small molecules, or pathogens (Ko et al., 2009). For example, a three-way “triangle” model, correlating genotype, gene expression, and phenotype data, has been devised to identify genetic variants that contribute to chemotherapeutic-induced cytotoxicity through their effects on gene expression (Huang et al., 2007b). Nevertheless, quantifying the significance of a finding from such an integrative approach remains to be fully addressed.

## Materials and Methods

### Functional integration

**Figure 1:**
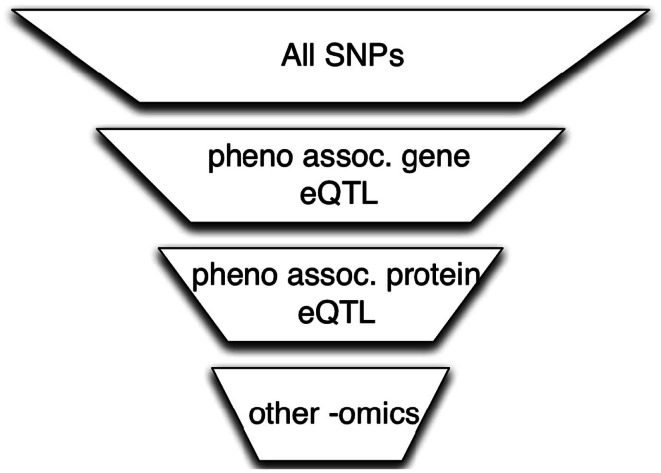
**The use of omics data to perform SNP filtering**. A series of functional filters is applied to the original SNP set to perform data dimensional reduction. The reduced multiple testing burden may improve power to detect genotype associations with phenotype.

A simple approach to integrate high-throughput functional datasets (e.g., from studies of the transcriptome, proteome, or microRNAome) with genome-wide genotype data obtained from microarray- or sequencing-based studies is to select SNPs that meet certain functional criteria as illustrated in the example in Figure 1. In the first step of this example, SNPs are filtered by requiring that they be associated with genes whose expression levels are associated with the phenotype (Zhong et al., 2010). In the next step, we further reduce the number of SNPs by requiring that they be associated with protein levels that are themselves associated with the phenotype. This process can continue using other omics datasets.

**Figure 2:**
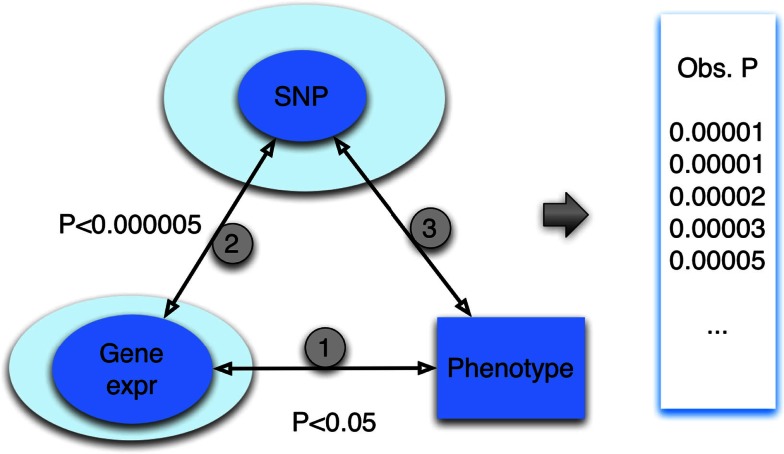
**The “triangle” method for integration of genotype, gene expression, and phenotype data**. Through a series of steps, heterogeneous datasets, involving SNPs, gene expression and trait are integrated. At each step, a *p*-value threshold is applied. In general, the *p*-value threshold used is arbitrary; in practice, the choice allows for prioritization of genes or SNPs. The result of the triangle method is a set of SNP association *p*-values (represented by the “obs *p*” in the figure).

To simplify the description, we focus on the case in which only the gene (mRNA) expression data are integrated, which is depicted with the diagram in Figure 2. This triangle approach and variations thereof were proposed by Huang et al. (2007b) and others (Zhong et al., 2010) and applied to an array of cellular phenotypes. The first step of this method aims to identify a set of gene expression traits associated with the given phenotype at an arbitrarily set *p*-value threshold, *p* < *p*_gene-phenotype_. It is important to emphasize that this threshold, as in the subsequent thresholds to be defined below, is generally set arbitrarily. In practice, these thresholds are used to prioritize genes or SNPs for downstream analyses. Indeed, one aim of our study is to quantify the significance of an association from a triangle method regardless of the choice of thresholds used during the integrative process. The second step of the method is to identify SNPs that are associated with the selected gene expression traits again at an arbitrarily set threshold, *p* < *p*_SNP-gene_. At a stringent threshold, this step maps the gene expression traits to genomic loci; this step thus identifies the eQTLs for the corresponding genes. Finally, in the last step of the triangle, the resulting SNPs are interrogated for association with the phenotype. Our primary aim is to describe a method to quantify the significance of the SNPs resulting from this multi-step “triangle” approach.

### Naïve FDR of selected SNPs

**Figure 3:**
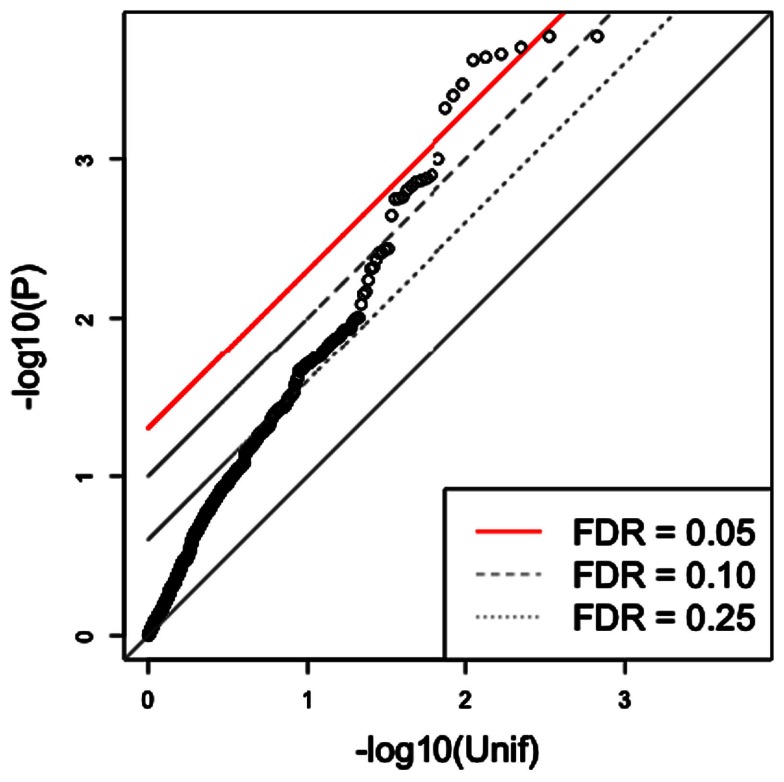
**Traditional FDR applied to the triangle method for simulated data**. The triangle method may yield numerous highly significant SNP associations, based on the traditional FDR approach, from a simulated phenotype. In this case, several significant SNPs are obtained from simulated data although none should be observed.

Since the triangle method is a multi-step approach that derives a final SNP set from a series of (potentially) increasingly stringent thresholds, it is reasonable to expect that such an approach should yield a final set with substantially reduced false discovery rates (FDRs) for association with the phenotype. A simple approach to assess the significance of the findings for this subset of SNPs would be to compute the FDR for them (Storey and Tibshirani, 2003). We illustrate the problem of this approach in Figure 3 in which we show the QQ plot of the associations after applying the triangle method to a simulated phenotype, which has no association with genotype. In this particular example, the first threshold *p*_gene-phenotype_ was set at 0.05 while *p*_SNP-gene_ was set at 5 × 10^−6^. Circles above the red line represent SNPs with FDR < 0.05. (Strictly speaking, circles with *p*-values less than the one with the largest *p*-value that goes above the red line has FDR < 0.05.) As the figure indicates, the triangle method may yield several spurious associations, if we rely on a “naïve” FDR approach. This example shows the need to develop a more sophisticated approach to estimate the significance of results in this integrative context.

### Simulating the null distribution

**Figure 4:**
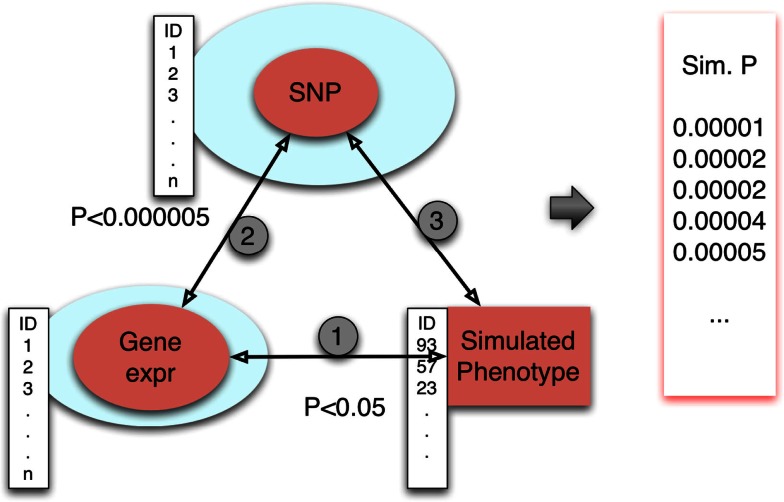
**Simulating the null distribution**. Simulated phenotypes were obtained by permuting phenotype values. Associations between gene expression traits and phenotype were done conditional on preserved correlation structure between the gene expression traits. The eQTLs for selected genes were retrieved from our eQTL database SCAN. Finally, the eQTLs were tested for association with phenotype. For each of *n* = 1000 replicates, a set of SNP association *p*-values is generated.

We describe here our approach to generating an empirical null distribution of *p*-values (Figure 4). First, let *Y*_1_, *Y*_2_, *Y*_3_, …, *Y_n_* be simulated phenotypes obtained from permuting the phenotype data. (Typically, *n* = 1000.) In case covariates are used, they should be relabeled in sync with the phenotype. For each simulated phenotype, we apply the same triangle method. For each *Y_i_*, we derive the set of gene expression traits *g_ij_* that meet the threshold, *p*-value < *p*_gene-phenotype_, where the associations between the phenotype *Y_i_* and gene expression traits are calculated while preserving the correlation structure of all gene expression phenotypes. For each *g_ij_*, we retrieve the set of eQTLs, *S_ijk_*, associated with the gene at the pre-defined threshold, *p*-value < *p*_SNP-gene_. The subset of these eQTL SNPs that satisfy *p*-value < *p*_SNP-phenotype_ provides a set of *p*-values $\left\{{\text{P}}_{ijk^{\prime }}\right\}$, for each simulated phenotype *Y_i_*. Note that each such set $\left\{{\text{P}}_{ijk^{\prime }}\right\}$ of *p*-values may differ in count between simulated phenotypes. Note that *i* indexes simulations, *j* indexes genes, and *k* indexes eQTLs.

We utilize these sets of *p*-values derived from simulated phenotypes to estimate the null distribution of *p*-values. Having shown the limitation of the use of the traditional FDR for the integrative triangle method, we derive a simple formula to estimate the FDR using this empirical null distribution.

### Empirical FDR

We closely follow Storey’s approach (Storey and Tibshirani, 2003) to estimate the FDR. The difference in our approach is that we do not assume that the null distribution of *p*-values is uniform. Instead, we use the empirical distribution generated by simulating the phenotype and performing the integrative analysis. We define the significance level *t* and reject the null hypothesis of no association for all *p*-values smaller than *t*. We use the actual values in the observed vector of *p*-values as cutoff. Thus, for each *p*-value, *t*, in the observed vector of *p*-values, we compute the FDR of the strategy of rejecting all *p*-values less than or equal to *t*. Let the number of falsely significant SNPs be denoted as *F*(*t*) = #{null*p_i_* ≤ *t*, *i* = 1, …, *m*} and the number of significant SNPs be denoted as *S*(*t*) = #{*p_i_* ≤ *t*, *i* = 1, …, *m*} with *m* the total number of SNPs after applying the integrative approach. We estimate the FDR as follows:

$$\begin{array}{rcll} FDR\left(t\right) & =E\left[\frac{F\left(t\right)}{S\left(t\right)}\right] & & \\ & \approx \frac{E\left[F\left(t\right)\right]}{E\left[S\left(t\right)\right]} & & \text{(1)}\\ & =\frac{mP\left(p\leq t\;\text{and null}\right)}{mP\left(p\leq t\right)} & & \\ & =\frac{P\left(p\leq t\;\text{and null}\right)}{P\left(p\leq t\right)}\; & & \text{(2)} \end{array}$$

where *E*[.] is the expectation operator. The approximate equality in Eq. 1 is proven by Storey (2003).

The denominator is estimated using the observed number of significant SNPs *p* ≤ *t*,

$$\#\left\{p_{\text{obs, }i}\leq \;t,\;i=1,\ldots ,\;m\right\}/m$$

**Figure 5:**
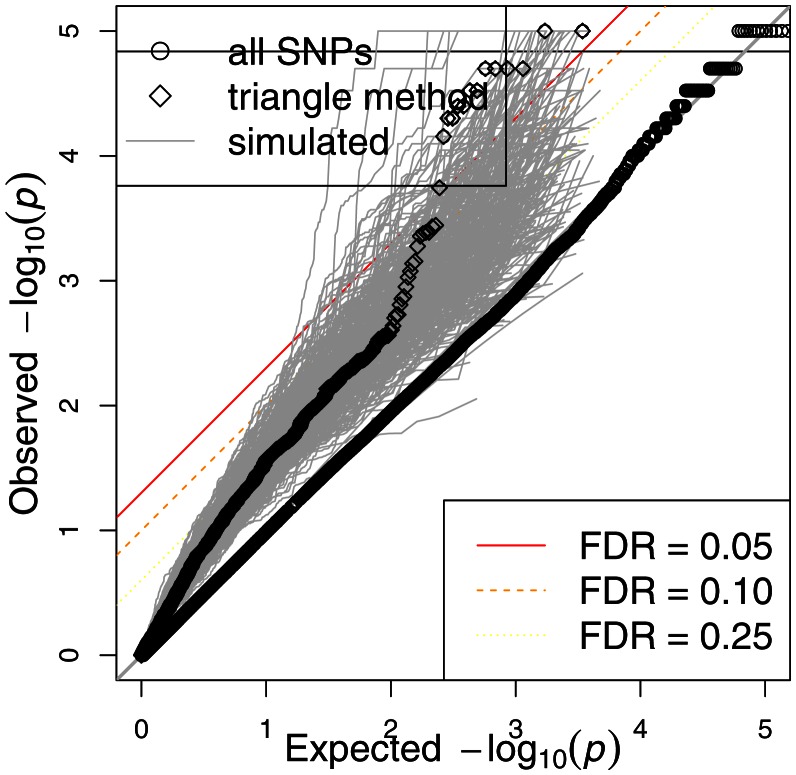
**Distribution of *p*-values for all SNPs and triangle method-derived SNPs from actual phenotype as well as simulated phenotypes**. QQ plots are shown for all SNPs as well as the SNPs that come of the triangle method. Gray dots represent the QQ plots for the triangle method-derived SNPs from 1000 simulated phenotypes. Note that the triangle method may yield spurious associations if we rely on the traditional FDR.

The numerator can be factored as *P*(*p* ≤ *t* and null) = *P*(*p* ≤ *t* | null)·*P*(null). The first factor *P*(*p* ≤ *t* | null) is estimated using the empirical distribution: #{*p*_sim,*i*_ ≤ *t*, *i* = 1, …, *M*_0_}/*M*_0_ where the *p*_sim_’s are the *p*-values generated with the simulated phenotypes and *M*_0_ is the sum (across all simulations, *M*_0_ = Σ*m_o,s_*, where *m_o,s_* corresponds to the number of eQTLs selected after applying the triangle method to the simulated phenotype *Y_s_*) of the total number of SNPs selected using the simulated phenotypes. Note that for uniformly distributed *p*-values, we would have *P*(*p* ≤ *t* | null) = *t*. We know, however, that when the set of SNPs are derived from the integrative approach, the null *p*-values may not be distributed uniformly, as illustrated in Figure 5. The second factor *P*(null) is the proportion of SNPs that are unrelated to the phenotype and may be estimated as the ratio

$${\hat{\pi }}_{0}=\frac{\#\left\{p_{\text{obs, }i\;}>\;t,\;i=1,\;\ldots ,\;m\right\}/m}{\#\left\{p_{sim,\;i\;}>\;t,\;i=1,\ldots ,M_{0}\right\}/M_{0}}$$

or may be set to 1 to yield a more conservative estimate of FDR.

In summary, we estimate the FDR based on the empirical distribution as follows:

$$\begin{array}{rcll} \text{e}\hat{\text{FDR}}\left(t\right) & =\frac{\#\left\{p_{\text{sim}}\leq t\right\}{/M}_{0}}{\#\left\{p_{\text{obs}}\leq t\right\}/M}.\frac{\#\left\{p_{\text{obs}}>\lambda \right\}/M}{\#\left\{p_{\text{sim}}>\lambda \right\}{/M}_{0}} & & \text{(3)}\\ & =\frac{\#\left\{p_{\text{sim}}\leq t\right\}}{\#\left\{p_{\text{sim}}\leq t\right\}}.\frac{\#\left\{p_{\text{obs}}>\lambda \right\}}{\#\left\{p_{\text{sim}}>\lambda \right\}} & & \text{(4)} \end{array}$$

with λ = 0.5. We can also use the more conservative estimate

$$\text{e}\hat{\text{FDR}}\left(t\right)\leq \frac{\#\left\{p_{\text{sim}}\leq t\right\}{/M}_{0}}{\#\left\{p_{\text{obs}}\leq t\right\}/M}$$

In order to ensure increasing FDR for increasing *p*-values, we define *q*-values as

(5) $$\hat{q}\left(t\right)=\text{mi}{\text{n}}_{p\geq t}\text{e}\hat{\text{FDR}}\left(p\right)$$

### Etoposide pharmacogenomics

A triangle method, similar to the one described here, had been originally applied to cellular sensitivity data for etoposide (Huang et al., 2007b), one of the most widely used anti-cancer agents. Using our empirical FDR approach, we re-analyzed the same phenotype data from the original experiments, which had sought to quantify the cytotoxic effect of the drug on the cell lines using a colorimetric-based assay, as previously described (Huang et al., 2007b). We conducted our study on the 90 HapMap cell lines of European descent (CEU). The quantitative trait used here was IC_50_, defined as the concentration required for 50% cell growth inhibition.

## Results

### R function for calculating empirical FDR

We provide an R function for estimating the empirical FDR that can be used once the observed and the simulated *p*-values are generated (http://www.scandb.org/newinterface/empiricalFDR.R). The way these *p*-values are generated will depend on the specific integration method used, the eQTL mapping database, and the number of components in the “genetic machinery.”

#### Computation time

For step 1 (see Figure 4) we need to compute about 10,000 (the number of transcripts) linear regressions. This can be achieved in a few seconds using R and the fast linear regression computation in R such as implemented by us and made available in http://www.scandb.org/newinterface/fastlm.R. For step 2, we only need to query the eQTLs for the new set of genes from step 1, which takes a fraction of a second. For step 3: after applying steps 1 and 2 only a few SNPs are left (typically around 1000 or less). This can also be done in a fraction of a second. Adding up all three steps, the method with 1000 permutations would take a couple of hours of computing time on a typical desktop available in 2012.

### Traditional GWAS and SNP selection via eQTLs

The GWAS of etoposide IC_50_ did not yield any significant signals, as perhaps expected from the small sample size. Figure 5 shows a QQ plot of the distribution of *p*-values (as circles). However, we found a highly significant enrichment for gene regulatory signals among the etoposide-associated variants relative to frequency-matched SNPs (Gamazon et al., 2010a). This finding raises the possibility of the use of eQTL annotation to increase the power to detect true associations. We therefore proceeded to incorporate eQTL functional annotation through the integrative triangle method.

### Genetic variation associated with etoposide cytotoxicity identified through the triangle method

Expression levels had been generated by our group on these cell lines for more than 10,000 genes, allowing us to perform associations between etoposide IC_50_ and gene expression traits; those genes meeting *p* < 0.05 (see Table S1 in Supplementary Material) were carried forward in the triangular analysis. We then utilized SCAN (Gamazon et al., 2010b), a public repository for the results of our eQTL studies on the HapMap cell lines, to annotate the selected genes showing association with etoposide IC_50_ with expression-associated SNPs (*p* < 10^−4^; see Table S2 in Supplementary Material). Finally, the selected eQTLs were tested for association with etoposide IC_50_. Figure 5 shows the QQ plot of association *p*-values for all SNPs, the QQ plot for the final SNP set derived from the triangle method, and the QQ plot for the triangle method-prioritized SNPs from each of 1000 simulated phenotypes. The figure illustrates that certain eQTL SNPs from this triangle method-derived SNP set attained a (traditional) FDR < 0.05, but also that the triangle method may yield spurious associations using the traditional FDR.

### Empirical FDR identifies significant associations with cellular sensitivity to etoposide

We applied our proposed empirical FDR method to the observed set of *p*-values from the triangle analysis-derived set of SNPs. To generate an empirical null distribution of *p*-values, we conducted simulations (see Materials and Methods). Table S3 in Supplementary Material lists the most significant etoposide-associated SNPs based on our empirical FDR method. Note the comparison between traditional FDR and eFDR for the most highly ranked SNPs prioritized by the triangle method (based on unadjusted *p*-value), showing that traditional FDR inflates the significance of selected SNPs.

## Discussion

Integrative approaches to diverse genomics datasets promise to resolve some important biological problems and, perhaps as importantly, generate novel hypotheses. Here we developed a *computationally feasible* permutation method to *quantify the significance* of findings arising from an integrative approach. The triangle method, a highly plausible approach to SNP prioritization and an example of how diverse high-throughput datasets may be integrated, requires an assessment of the resulting findings. This integrative method incorporates genotypic and expression data to identify trait-correlated genes that are under the regulation of eQTLs, yielding a set of candidate SNPs potentially important for the genetic etiology of the trait. Our proposed empirical FDR approach not only takes into account the integrative nature of the triangle method, but the approach also accounts for the correlation structure among gene expression traits and among genotypes. Our empirical FDR approach aims to provide a sound quantification of the significance of the prioritized SNPs from the integrative method.

It should be noted that our approach separates the phenotype from what we are calling the “genetic machinery” (e.g., genotype, gene expression, protein expression, methylation). Only the phenotype is permuted and the relationships within the genetic machinery are preserved. Consequently, we avoid having to perform multiple eQTL mappings (the most computationally costly permutation) because *p*-values in each arm are used for prioritization and not for determining the significance of the associations. Importantly, our approach differs from other approaches wherein the permutation is conducted on each arm of the triangle. In the latter approach, the threshold for significance can be arbitrary or unnecessarily conservative. A well-chosen set of thresholds will determine the performance of the integrative approach. In our method, we provide a measure of significance that is well-calibrated regardless of the set of thresholds used. Furthermore, in contrast to approaches that apply a threshold (e.g., Bonferroni) at each step of the integrative process, our method provides an overall measure of significance for the results of the integrative analysis.

Our quantification approach can easily accommodate hub eQTL analysis (SNPs associated with multiple genes, also referred to as master regulators). In the filtering procedure we require that the SNPs be eQTLs for a number of phenotype-associated gene expression traits. As long as the permutation steps follow the same filtering algorithm as the one used for the observed data, our method will yield the right FDR. Likewise, our method can be applied to both quantitative and binary outcomes.

In this study, we also explored the limitations of the traditional FDR when applied to an integrative approach such as the triangle method. In particular, we found that traditional FDR may yield spurious associations from simulated phenotypes. Furthermore, while the use of eQTL information may improve power to detect true associations, traditional FDR may still inflate the significance of the selected SNPs.

We applied our empirical FDR approach to a study of cellular sensitivity to etoposide. Etoposide is a topoisomerase II inhibitor (Sinha et al., 1988) widely used against lung cancer, non-Hodgkin’s lymphoma, myelogenous leukemia, and Kaposi’s sarcoma. As in the case of other chemotherapeutic agents, the drug is associated with serious toxicities, including bone marrow suppression, diarrhea, and fatigue as well as treatment-induced acute myeloid leukemia (Mistry et al., 2005). Thus, the identification of predictors of response or potentially debilitating toxicities associated with etoposide, including genetic variations, is key to the implementation of an effective treatment regimen and, longer-term, to the realization of an individualized approach to therapy. Based on cell lines derived from large pedigrees, it has been reported that a significant genetic component contributes to cellular sensitivity to etoposide (Peters et al., 2011).

Here, using our empirical FDR method, we identified 12 SNPs showing significant association (eFDR < 0.15) with cellular sensitivity to etoposide through their effect on gene expression. The 12 SNPs represent four independent genomic loci (on chromosome 8q12, 2p24, 10q23, and 16q24), of which the 10q23 SNPs are located in the glutamate receptor ionotropic delta-1 subunit (*GRID1*) gene. The expression target genes of rs9808546 (on chromosome 2) show a highly significant enrichment for *acetylation* [*n* = 27, Benjamini–Hochberg (BH) FDR = 0.0018] and *phosphoprotein* (*n* = 51, BH FDR = 0.0027; Huang da et al., 2009), consistent with studies that have shown that histone deacetylase inhibitors sensitize cells to the cytotoxic effects (particularly) of topoisomerase II agents such as etoposide (Kurz et al., 2001; Marchion et al., 2004; Hajji et al., 2008, 2010). Importantly, having provided a sound quantification of the significance of the genotype-phenotype associations, the gene expression targets of the identified eQTLs provide a set of candidate genes for functional validation and a plausible mechanism for how the genetic variation may mediate their phenotypic effect.

The R code we provide can be used to compute the empirical FDR for any case in which empirical null *p*-values are available regardless of the method used to generate them. Thus, it should prove useful for other integrative approaches.

In case there are confounding factors that yield more gene expression traits associated with the phenotype, our method yields a conservative estimate of FDR. The effect of the confounders is to increase the number of noisy genes in the first step and consequently to generate more null eQTLs than there should be in the final set. This fact decreases the overall significance of real associations and our method still provides an unbiased estimate of the significance.

In summary, we have developed a computationally feasible approach to assess the significance of genotype-phenotype associations prioritized by an integrative genomic method. As omics datasets become routinely integrated to address important biological problems, the issue our study sought to address becomes increasingly more relevant.

## Conflict of Interest Statement

The authors declare that the research was conducted in the absence of any commercial or financial relationships that could be construed as a potential conflict of interest.

## Supplementary Material

The Supplementary Material for this article can be found online at http://www.frontiersin.org/Statistical_Genetics_and_Methodology/10.3389/fgene.2012.00202/abstract

Supplementary Table S1 **Top gene expression-trait correlations**.

Supplementary Table S2 **Top SNPs from etoposide GWAS**.

Supplementary Table S3 **Top associations from the eFDR approach**.
